# Landscape of T Cells Transcriptional and Metabolic Modules During HIV Infection Based on Weighted Gene Co-expression Network Analysis

**DOI:** 10.3389/fgene.2021.756471

**Published:** 2021-09-16

**Authors:** Jianting Xu, Jiahui Pan, Xin Liu, Nan Zhang, Xinyue Zhang, Guoqing Wang, Wenyan Zhang

**Affiliations:** ^1^ Institute of Virology and AIDS Research, The First Hospital of Jilin University, Changchun, China; ^2^ College of Basic Medicine, Jilin University, Changchun, China; ^3^ College of Mathematics, Jilin University, Changchun, China

**Keywords:** HIV infection, T cell, transcriptional modules, metabolomics, weighted gene co-expression network analysis

## Abstract

Human immunodeficiency virus (HIV) causes acquired immunodeficiency syndrome (AIDS). HIV infection affects the functions and metabolism of T cells, which may determine the fate of patients; however, the specific pathways activated in different T-cell subtypes (CD4^+^ and CD8^+^ T cells) at different stages of infection remain unclear. We obtained transcriptome data of five individuals each with early HIV infection, chronic progressive HIV infection, and no HIV infection. Weighted gene co-expression network analysis was used to evaluate changes in gene expression to determine the antiviral response. An advanced metabolic algorithm was then applied to compare the alterations in metabolic pathways in the two T-cell subtypes at different infection stages. We identified 23 and 20 co-expressed gene modules in CD4^+^ T and CD8^+^ T cells, respectively. CD4^+^ T cells from individuals in the early HIV infection stage were enriched in genes involved in metabolic and infection-related pathways, whereas CD8^+^ T cells were enriched in genes involved in cell cycle and DNA replication. Three key modules were identified in the network common to the two cell types: *NLRP1* modules*, RIPK1* modules, and *RIPK2* modules. The specific role of NLRP1 in the regulation of HIV infection in the human body remains to be determined. Metabolic functional analysis of the two cells showed that the significantly altered metabolic pathways after HIV infection were valine, leucine, and isoleucine degradation; beta-alanine metabolism; and PPAR signaling pathways. In summary, we found the core gene expression modules and different pathways activated in CD4^+^ and CD8^+^ T cells, along with changes in their metabolic pathways during HIV infection progression. These findings can provide an overall resource for establishing biomarkers to facilitate early diagnosis and potential guidance for new targeted therapeutic strategies.

## Introduction

Acquired immunodeficiency syndrome (AIDS) is caused by human immunodeficiency virus (HIV) infection (Sepkowitz, 2001), a lentivirus belonging to the subgroup of retro-RNA viruses. HIV infection induces changes in T lymphocyte functions, leading to alterations in the entire immune system of the host and disruption of homeostasis. These hallmarks of HIV infection manifest differently based on the infection period (Weiss, 1993).

As a component of the host’s immune defence system, T cells participate in a series of immune responses against HIV infection (Gupta and Saxena, 2021). Both CD4^+^ and CD8^+^ T cells participate in the host adaptive immune response against bacterial and viral infections. In particular, CD4^+^ T cells can “help” the activity of other immune cells by releasing cytokines and small protein mediators, whereas CD8^+^ T cells directly kill the target cells after activation in the human body (Hoyer et al., 2014). HIV mainly infects CD4^+^ T lymphocytes. Clinically, HIV infection results in low blood CD4^+^ T-cell levels. In addition, CD4^+^ T cells directly inhibit HIV by promoting other T cells to resist viral infection (Johnson et al., 2015). CD8^+^ T cells are widely distributed on the surface of inhibitory and cytotoxic T lymphocytes, and their kinetics differ from those of CD4^+^ T cells during HIV infection (Xu et al., 2014). However, the overall molecular mechanisms underlying the changes and actions of CD4^+^ and CD8^+^ T cells after HIV infection remain to be elucidated.

Moreover, changes in metabolism also represent a key to understanding the immune response during pathogen invasion. Metabolism plays a fundamental role in supporting the growth, proliferation, and activation status of T cells (Palmer et al., 2016; Masson et al., 2017). For example, CD8^+^ T cells increase oxidative phosphorylation and steadily increase the glycolysis rate, whereas CD4^+^ T cells reduce fatty acid oxidation (Bantug et al., 2018). In HIV-1 infection, changes in cell metabolism affect the susceptibility of CD4^+^ T cells; HIV-infected CD4^+^ T cells exhibit elevated metabolic activity and metabolic potential compared with those of HIV-exposed but uninfected cells (Valle-Casuso et al., 2019). Detection of gene expression changes related to metabolism could provide insight into changes in metabolic pathway activity under HIV infection (Lee et al., 2012).

Weighted Correlation Network Analysis (WGCNA) is a method that can be used to analyze highly correlated gene modules in multiple samples and discover the relationship between the modules and specific functions (Langfelder and Horvath, 2012). It can provide panoramic information of T cell transcriptome modules after HIV infection. We performed WGCNA to assess changes in gene expression profiles in human CD4^+^ T cells and CD8^+^ T cells during HIV-1 infection. We then focused on acute HIV infection to explore the possible antiviral effects of the two cell types after their interaction with HIV-1 and to identify some key pathways and targets involved in the infection response. Ultimately, this study can highlight the metabolic changes occurring in T cells at different stages of HIV infection using the metabolic algorithm. These findings can show the panoramic transcription and metabolism modules and provide new insights for further biomarker discovery. This research can facilitate the early detection of HIV infection and ultimately the development of new strategies for effective infection control.

## Materials and Methods

### Data Collection

We first searched the GEO database GSE6740 and downloaded the gene expression profiles from five individuals each with acute HIV infection, chronic progressive HIV infection, and no HIV infection. These data were reported and deposited to the GEO database by Hyrcza et al. (2007).

### WGCNA and Module Recognition

WGCNA is a widely used data-mining method in genomic applications. We used the WGCNA software package in the R environment (R Foundation for Statistical Computing, Vienna, Austria) to construct a co-expression network of differentially expressed genes between HIV-infected and non-infected individuals. The algorithms were used to calculate the correlations between the levels of differentially expressed genes after the selection of an appropriate threshold (*β*), and then a scale-free network was constructed. We used the minimum value of *β* greater than 0.85 as the most suitable threshold, and then used the topological overlap matrix (TOM) (direct correlation + indirect correlation) between genes for hierarchical clustering to construct a clustering tree, which contained different gene modules represented by different colours. In this process of module identification, we set the minimum number of genes contained in the module to 50 (Zhang and Horvath, 2005).

### Metabolic Pathway Activity Analysis

The number of metabolic genes enriched in a particular pathway was combined with the expressional values of the genes using the following formulas:

$$E_{ij}=\frac{\sum_{k=1}^{n_{j}}gik}{n_{j}}$$ (1)

where *E*_*ij*_ indicates the average expression level of the *i*th gene in the *j*th cell type, *g*_*ik*_ indicates the expression level of the *i*th gene in the *k*th sample, and *n*_*j*_ indicates the number of samples in the *j*th cell type;

$$r_{ij}=\frac{E_{ij}}{\frac{1}{N}\sum_{j}^{N}E_{ij}}$$ (2)

where *N* indicates the number of cell types, and *r*_*ij*_ represents the ratio of the average expression level of the *i*th gene in the *j*th cell type to the average level of the gene in all cell types. A ratio greater than 1 indicates that the gene expression level in the cell is higher than the average expression level in all cells, and a ratio below 1 indicates the opposite pattern; and

$$S_{tj}=\frac{\sum_{i=1}^{m_{t}}W_{i}\times r_{ij}}{\sum_{i=1}^{m_{t}}W_{i}}$$ (3)

where *s*_*tj*_ indicates the score value of the *t*th pathway in the *j*th cell type (the higher the score, the stronger the significance), *m*_*t*_ indicates the number of genes in the *t*th pathway, and *W*_*i*_ represents the weighted value of the *i*th gene (the reciprocal of the *t*th pathway metabolic gene) (Xiao et al., 2019).

### Functional Annotation and Protein-Protein Interaction Network

Using clusterProfiler (an R package), we performed pathway enrichment analysis of the differentially expressed genes with respect to Gene Ontology terms (GO) and Kyoto Encyclopedia of Genes and Genomes (KEGG) pathways, using default parameters. We then constructed a protein-protein interaction network based on these data using the STRING database in Cytoscape version 3.8.2. We also identified the chromosomal localisation of all genes in the target pathway by using the Ensembl and Genecards websites.

## Results

### WGCNA Identification of Genetic Modules

In our study, the sample characteristics were divided into three stages as follows: Healthy, HIV acute infection, and HIV chronic infection. Then we choose the appropriate threshold (Supplementary Figure S1). We combined the relevant traits and modules of the sample for joint analysis to show the correlation between modules and traits using WGCNA’s systems biology method. Different modules were represented by different colours. Each module contained a set of highly connected genes, and the genes in each module might participate in similar pathways or have the same biological functions. These modules ranged from large to small according to the number of genes that they contained.

**FIGURE 1:**
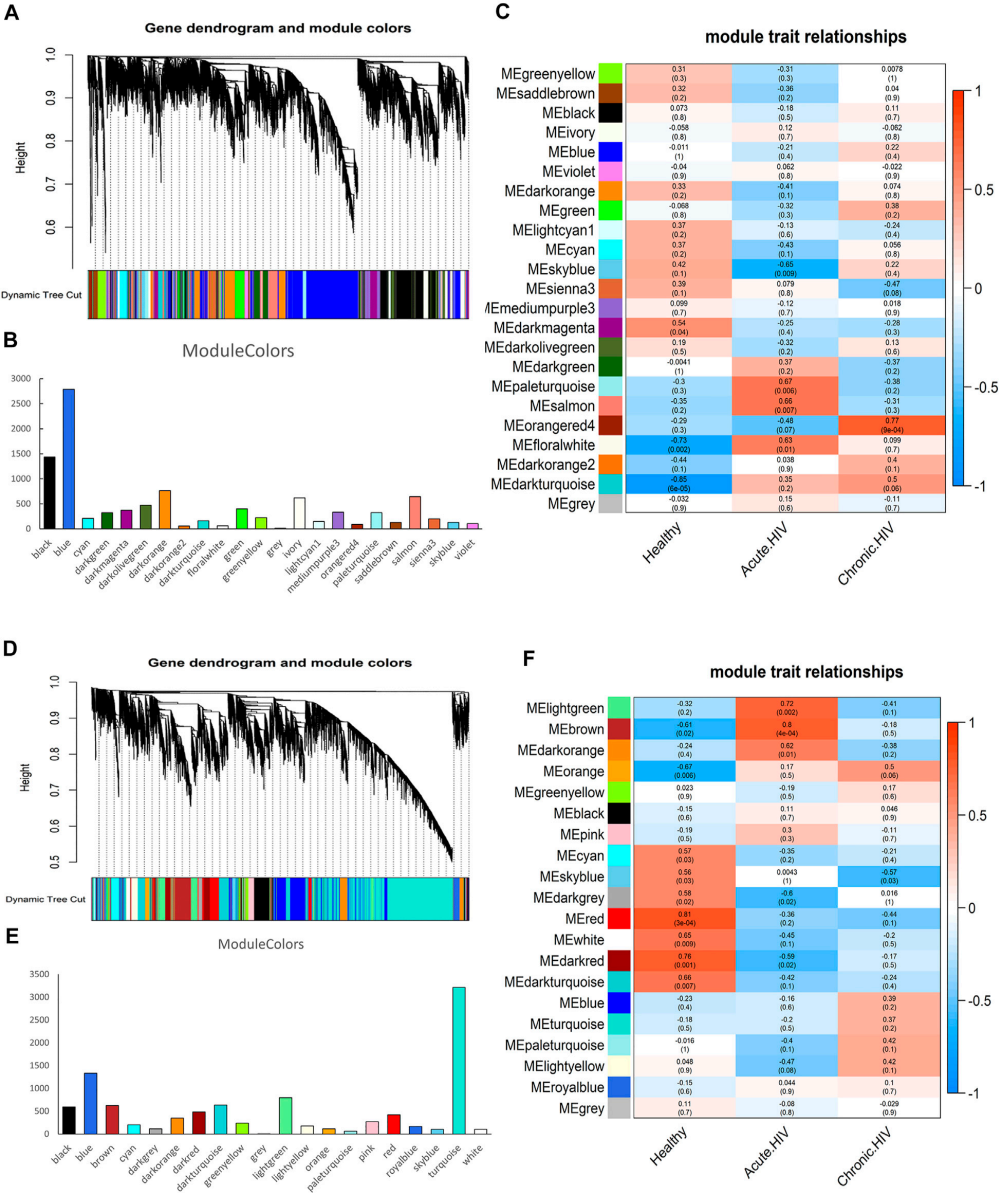
Identification of the co-expressed gene modules. **(A)** Hierarchical clustering dendrogram of the co-expressed gene modules in CD4^+^ T cells. **(B)** The number of genes contained in 23 modules of CD4 cells. **(C)** Correlation coefficients of module-trait relationships in CD4^+^ T cells at different infection periods are indicated by different colours. **(D)** Hierarchical clustering dendrogram of the co-expressed gene modules in CD8^+^ T cells. **(E)** The number of genes contained in 20 modules of CD8 cells. **(F)** Correlation coefficients of module-trait relationships in CD8^+^ T cells at different infection periods are indicated by different colours.

The results of co-expression network analysis are shown in Figure 1. The number of genes in the module is shown in Figures 1B,E and Supplementary Table S1. The correlation coefficient of each module is shown in Figures 1C,F. We identified 23 co-expressed gene modules for CD4^+^ T cells (Figure 1A). In terms of the number of genes contained in the module, MEblue was the largest module, containing 2,786 genes, whereas MEgrey was the smallest module, containing 13 genes. From the perspective of the correlation coefficient after infection, the module with the largest negative correlation coefficient was MEskyblue, with a value of −0.65; the module with the largest positive correlation coefficient was MEorangered4, with a value of 0.77. Each module has different functions. For example, Leukocyte transendothelial migration and FoxO signaling pathway are enriched in the MEskyblue.

We confirmed 20 co-expressed gene modules in CD8^+^ T cells (Figure 1D). From the perspective of the number of genes contained in the module, MEturquoise was the largest module, containing 3,214 genes; MEgrey was the smallest module, containing only one gene. From the perspective of the correlation coefficient after infection, the module with the largest negative correlation coefficient was MEdarkgrey, with a value of −0.6; the module with the largest positive correlation coefficient was MEbrown, with a value of 0.8. In addition, the coefficient of the MElightgreen ranked second, reaching 0.72. RNA transport and Viral carcinogenesis are enriched in the MElightgreen.

### Key Module-Activated Cell Processes

**FIGURE 2:**
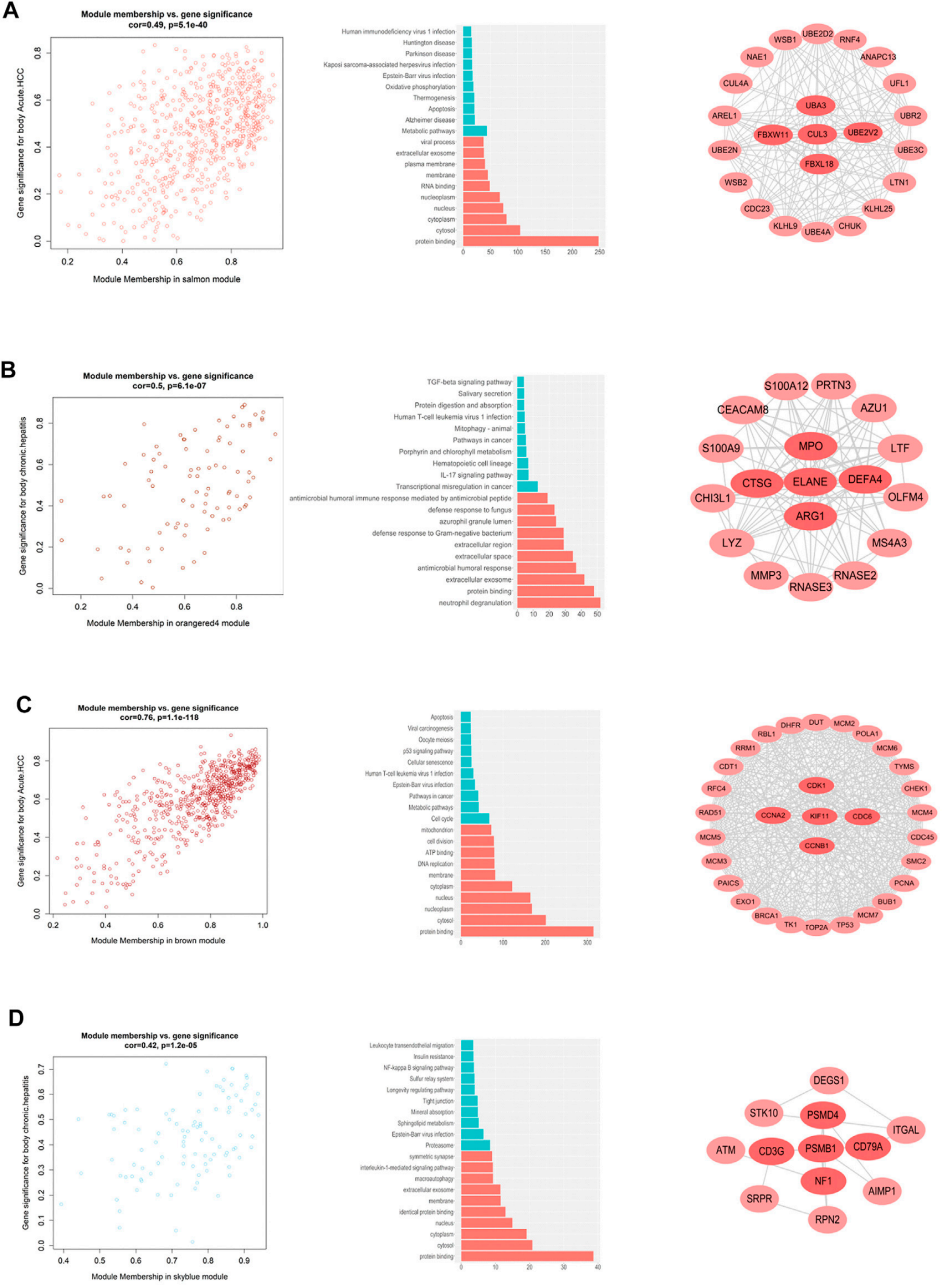
Co-expressed gene modules that interact with other genes in different stages of HIV infection. **(A)** Distribution of genes in the salmon module, GO and KEGG pathway enrichment, and key genes in the early HIV infection stage of CD4^+^ T cells. **(B)** Distribution of genes in the orange4 module, GO and KEGG pathway enrichment, and key genes of CD4^+^ T cells in the chronic HIV infection stage. **(C)** Distribution of genes in the brown module, GO and KEGG pathway enrichment, and key genes in the early HIV infection of CD8^+^ T cells. **(D)** Distribution of genes in the sky blue module, GO and KEGG pathway enrichment, and key genes in the chronic HIV infection of CD8^+^ T cells.

By calculating the correlation between the gene modules and the phenotype matrix, the key modules were screened out (i.e. those exhibiting higher correlations). The “salmon” module and the “orangered4” module were selected to represent the genes affected during early and chronic infection in CD4^+^ T cells, respectively, whereas the “brown” module and the “skyblue” module were selected to represent early and chronic infection in CD8^+^ T cells, respectively. We draw scatter plots of the relationship between gene saliency and module membership in the four modules respectively (Figure 2). Functional annotation of the key modules showed a distinct biological significance bias for each module (Supplementary Table S2) for example, the “salmon” module (early infection of CD4^+^ T cells) was significantly enriched in inactivation of metabolic and infection-related pathways (Figure 2A), whereas the “orangered4” module (chronic infection of CD4^+^ cells) was most significantly enriched in the TGF-beta signaling pathway and IL-17 signaling pathway, among others (Figure 2B). By contrast, the cell cycle and DNA replication were activated in the early infection CD8^+^ T cells (Figure 2C), whereas the proteasome and sphingolipid metabolism were largely activated in CD8^+^ T cells in the chronic infection stage (Figure 2D).

### Critical Pathways During Acute HIV Infection

**FIGURE 3:**
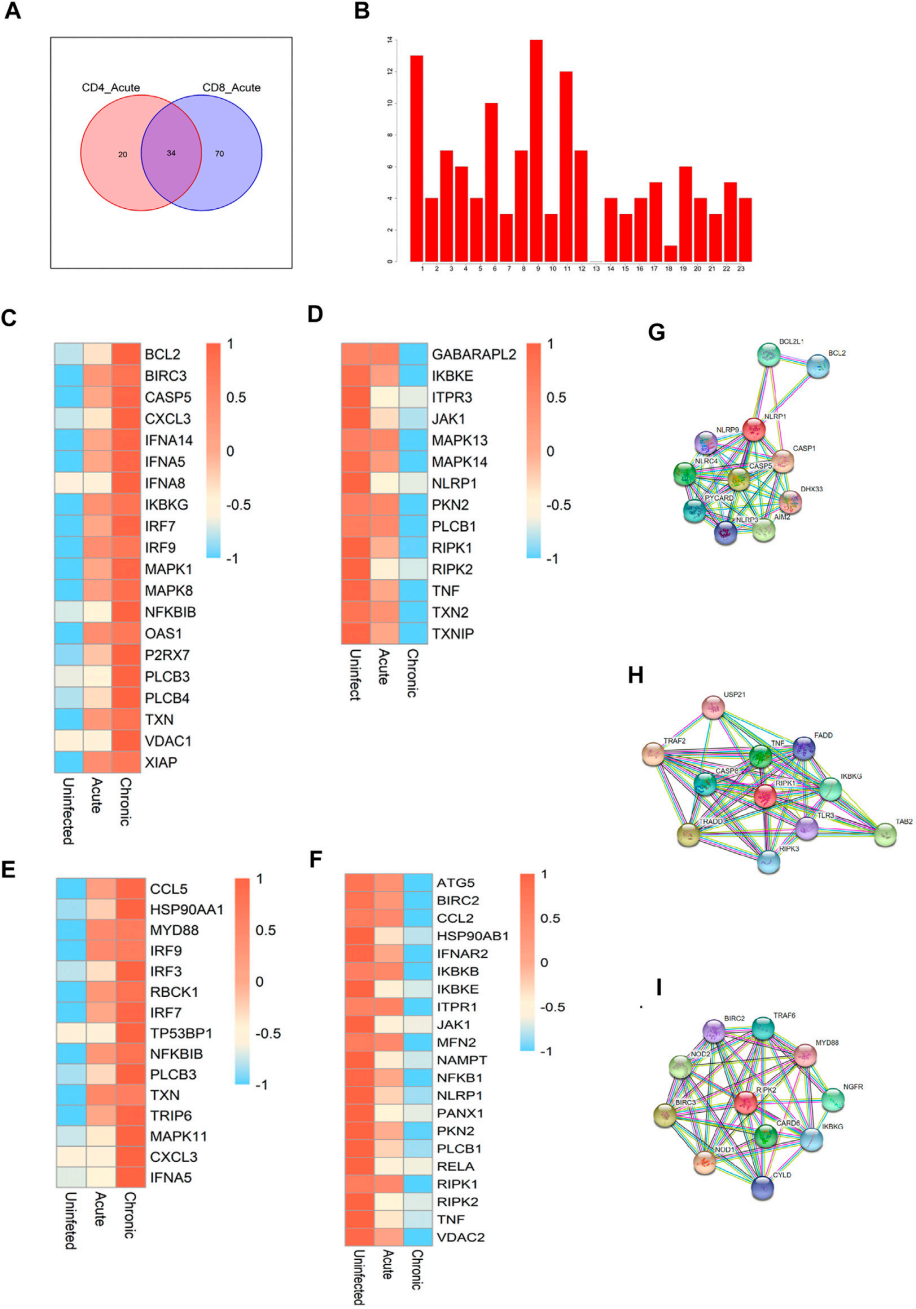
Critical pathways during acute HIV infection. **(A)** Enrichment of pathways in CD4^+^ and CD8^+^ T cells during the early stage of HIV infection. The Venn diagram shows a total of 34 pathways common to the two cell types, with 20 CD4^+^ T cell-specific pathways and 70 CD8^+^ T cell-specific pathways. **(B)** Chromosomal localisation of all genes in the NOD-like receptor pathway. **(C,D)** Gene modules and co-upregulated gene modules in the NOD-like receptor pathway in CD4^+^ T cells during different infection periods. **(E,F)** Co-upregulated and co-downregulated gene modules in CD8^+^ T cells during different HIV infection periods. **(G–I)** Protein-protein interaction network centralised with respect to NLRP1 **(G)**, RIPL1 **(H)**, and RIPK2 **(I)**.

We focused on the similarities and differences in the activated gene pathways between CD4^+^ and CD8^+^ T cells during early infection. There were 20 special pathways among CD4 cells and 70 special pathways for CD8 cells. In total, 34 gene pathways (Figure 3A) overlapped between the two cell types. Among them, the top pathways mainly included the following: metabolic pathways, human immunodeficiency virus 1 infection, NOD-like receptor signaling pathway, and cAMP signaling pathway, among others. In addition to metabolic pathways and HIV infection pathways, two pathways with broad significance, NOD-like signaling pathways rank the top among other pathways. So we further focused on the NOD-like receptor (NLR) pathway. NLRs are type pattern recognition receptors for the host, which can recognise the pathogen-related molecular patterns of viruses to regulate antiviral innate immune signaling pathways, thereby regulating the innate antiviral immune response (Zheng, 2021). We first identified the chromosomal localisations for all of the genes enriched in the NLR pathway (Figure 3B), as well as the expression modules showing consistent direction of change (down- or up-regulated) in the two cells in the context of early and chronic infection (Figures 3C–F). The modules that were co-up-regulated in CD4 and CD8 cells after infection included IRF9, IRF7, PLCB3, CXCL3, and TXN, among others. The co-down-regulated modules we identified included JAK1, PKN2, TNF, NLRP1, RIPK1, and RIPK2, among others. Among them, three core modules were selected, including NLR family pyrin domain containing 1 (NLRP1), receptor-interacting serine/threonine kinase 1 (RIPK1), and RIPK2. By constructing a protein–protein interaction network, we identified the genes that might interact with each other in the three modules (Figures 3G–I).

Differential activation of cell metabolism between CD4^+^ and CD8^+^ T cells before and after HIV infection.

**FIGURE 4:**
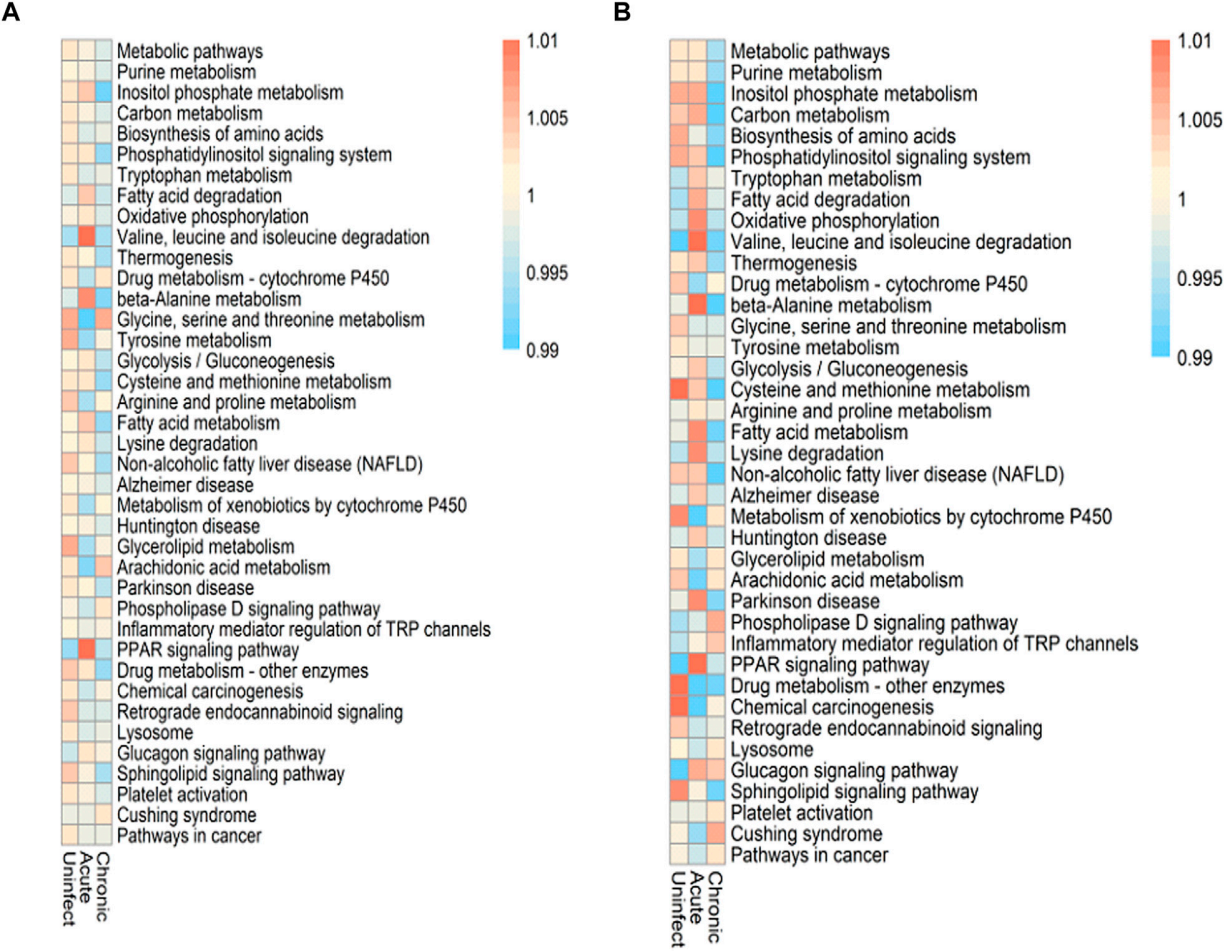
Altered metabolic pathways during HIV infection. **(A)** Changes and scores of the metabolic pathways in CD4^+^ T cells before and after infection and during different infection periods. **(B)** Changes and scores of the metabolic pathways of CD8^+^ T cells before and after infection and during different infection periods.

There are a total of 9,700 genes in our data set, and 1,352 genes related to metabolism are collected. These metabolic genes come from metabolic pathways. We selected 296 genes expressed by both as the most basic metabolic genes in this study. Since CD4^+^ and CD8^+^ T cells play different roles in the immune response, we assessed the differential activation and characteristics of these two cell types and screened their co-expressed metabolic genes. The cell metabolism pathways were differentially activated before and after infection. HIV infection also appears to disrupt the metabolic balance between these two cell types; some metabolic pathways were activated, whereas others remained unchanged. As shown in Figure 4, the pathways that were significantly altered in both types of cells in early infection included valine, leucine, and isoleucine degradation; beta-alanine metabolism; and PPAR signaling pathways. These three pathways also showed the greatest change in CD4^+^ T cells between the early infection and chronic infection stages. Among them, the PPAR signaling pathway also has significant changes in pathogen infections such as ZIKV and Neisseria meningitidis. More HIV-induced metabolic abnormalities were detected in CD8^+^ T cells compared with those occurring in CD4^+^ T cells. Before and after infection, the majority of pathways were changed in CD8^+^ T cells, along with some pathways such as the arachidonic acid metabolism pathway that was unchanged. In addition to the three most significant pathways mentioned above, the oxidative phosphorylation, fatty acid metabolism, and lysine degradation pathways also exhibited relatively large changes in CD8^+^ T cells between the acute and chronic infection stages. Thus, changes in these metabolic pathways may be conducive for these two cell types to cope with HIV infection.

## Discussion

The primary function of CD4^+^ T cells after HIV infection is related to DNA repair in response to DNA damage stimuli, along with positive regulation of cellular processes and other pathways, whereas CD8^+^ T-cell functions after infection are mainly related to cell mitosis, signal transduction, and transmission (Xu et al., 2014). In addition, network-based methods have been widely used in biological data analysis (Peng et al., 2021a; Peng et al., 2021b). Therefore, we use WGCNA analysis to find that CD4^+^ T cells from individuals in the early HIV infection stage were enriched in genes involved in metabolic and infection-related pathways, whereas CD8^+^ T cells were enriched in genes involved in cell-related changes, including the cell cycle and DNA replication. During chronic HIV infection, CD4 cells are mainly enriched in pathways related to immune defense, such as IL-17 signaling pathway. CD8 cells are mainly enriched in proteasome and sphingolipid metabolism. This finding identified many other pathways altered in the two T-cell subtypes at different stages of HIV infection. It also expands evidence for the field to enrich overall understanding of HIV infection-related gene alterations and modules. However, in chronic infection, the two types of cells share fewer pathways. Therefore, when screening critical pathways, we choose acute infection, which makes it easier to find infection markers and therapeutic targets.

In addition, few studies have focused on the systematic characteristics of the two cells at different stages of HIV infection or the similarities and differences between the two cells at the same stage. We used GO annotations and KEGG pathways to analyze the core pathways in the 2 T cell types during early and chronic HIV infection, and then explored key co-expression modules among them. We identified three key down-regulation modules: NLRP1 module, RIPK1 module and RIPK2 module. The central gene of the module represents the function of the module to a certain extent. Among them, RIPK1 and RIPK2 are the key mediators of cell apoptosis and death, as well as the inflammatory pathways (Festjens et al., 2007). RIPK1 and RIPK2 can be cleaved by HIV-1 protease, which affects important biological processes in the body such as host defence pathways and cell death (Wagner et al., 2015). However, the specific role of NLRP1 in the regulation of HIV infection in the human body remains to be determined. NLR is a type of germline-encoded pattern recognition receptor, which is mainly involved in the cytosolic sensing mechanism to detect viral infections in the body. NLRs participate in immune signaling pathways, including inflammasomes, nuclear factor -kappa B, and type I interferon signaling (Lupfer and Kanneganti, 2013). In terms of viral infection, NLRs play an impor tant role in both innate and adaptive immunity. NLRP1 was the first protein identified to form an inflammasome and is a sensor for a variety of pathogens, which can activate an antibacterial or antiviral immune response (Chavarria-Smith and Vance, 2015; Chavarria-Smith et al., 2016). RIPK family members have also been documented to be related to NLRs. The association of RIPK and NLRP1 in this study further confirms their role in HIV infection, although further experimental studies are needed to explore their actual link. Moreover, the core genes identified in each module, and the specific types of genes in the modules corresponding to different infection stages and cell types could guide new therapeutic targets of HIV infection.

Cellular immune metabolism has become one of the hottest research topics in immunology (Medzhitov, 2015). Previous studies also showed that HIV infection led to upregulation of amino acid metabolism, the tricarboxylic acid cycle, and fatty acid metabolism in human CD4^+^ T cells (Chan et al., 2007; Ringrose et al., 2008; Zhang et al., 2017). In our study, we utilised a novel algorithm to analyse differences in the metabolic pathways of CD4^+^ and CD8^+^ T cells before and after HIV infection. Our data demonstrate significant changes in three pathways of oxidative phosphorylation, fatty acid metabolism, and lysine degradation in CD8+T cells after early HIV infection compared with those assessed from individuals with chronic infection. The degree of metabolism of CD8 cells after infection is much stronger than that of CD4 cells. We have enriched the metabolic pathways of the two cells that are significantly altered in the early stage of HIV infection. These metabolic characteristics may be of great significance and warrant further investigation into identifying the mechanism of action of these two immune cell types after HIV infection.

In this study, we used WGCNA technology and metabolic algorithms to show a panoramic view of the core modules and metabolic pathways associated with HIV infection, providing new ideas and strategies for the development of HIV therapeutic targets and early diagnosis.

## Data Availability

Publicly available datasets were analyzed in this study. This data can be found here: GEO database—GSE6740.

## Supplementary Material

Supplementary Figure 1 Threshold filter. **(A)** The threshold of the module in CD4 cells is set to 9. **(B)** The screening of the module threshold in CD8 cells is set at 5.

Supplementary Table 1 Each module of the two types of cells contains the number of genes.

Supplementary Table 2 Key module functions. Two types of cell acute and chronic infections each have a key module, including KEGG enrichment and GO annotation.
