# Supplementary figures and images for: Landscape of T Cells Transcriptional and Metabolic Modules During HIV Infection Based on Weighted Gene Co-expression Network Analysis
Source: Front Genet. 2021 Sep 16;12:756471. doi: 10.3389/fgene.2021.756471 (PMC8481372; doi:10.3389/fgene.2021.756471)

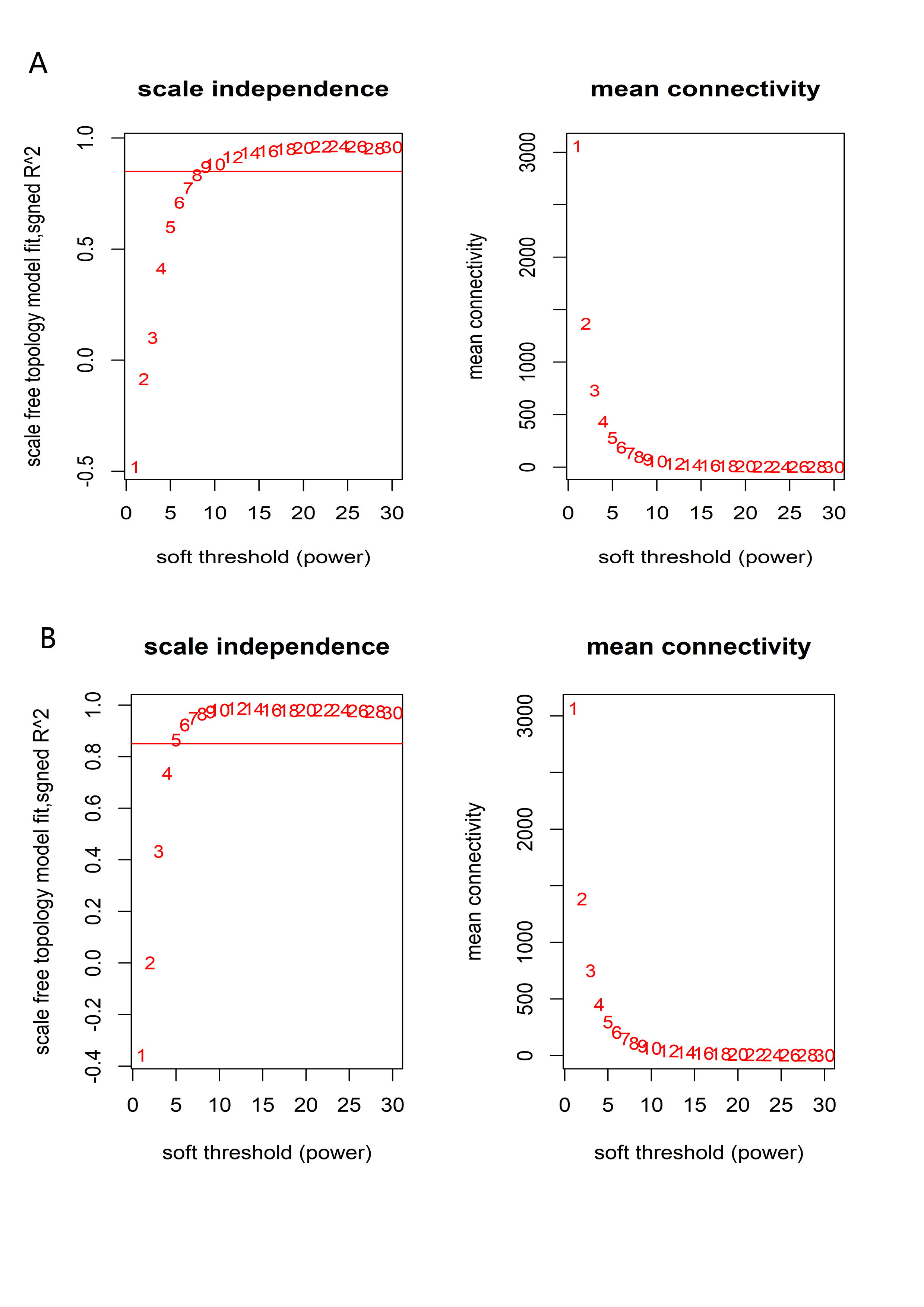

Supplement: Supplementary file 2 [file Image1.JPEG]
